# Supplementary material for: Expression of Castanea crenata Allene Oxide Synthase in Arabidopsis Improves the Defense to Phytophthora cinnamomi
Source: Front Plant Sci. 2021 Feb 15;12:628697. doi: 10.3389/fpls.2021.628697 (PMC7917121; doi:10.3389/fpls.2021.628697)
Supplement: Supplementary file 1 [file Data_Sheet_1.pdf]

SUPPLEMENTARY MATERIAL

Expression of *Castanea crenata* Allene Oxide Synthase in Arabidopsis Improves the Defense to *Phytophthora cinnamomi*

Serrazina et al.

	Wild type	CcAOS1	CcAOS2	CcAOS3
Rosette area (cm^2)	0.61 ± 0.14	0.71 ± 0.19 *	0.79 ± 0.30 *	0.83 ± 0.22 *
Root (dry weight; g)	0.0006±0.0002	0.0009±0.0005	0.0007±0.0005	0.0005±0.0002
Aerial (dry weight; g)	0.0049±0.0009	0.0069±0.0006 *	0.0098±0.0034 *	0.0078±0.0006 *
Time of flowering	73±6.67	81±14.67	94±6.0	100±0.00 *
Primary root length (cm)	2.28±1.22	3.30±1.02 *	2.77±1.14	3.55±1.38 *
Lateral root number	6.4±3.5	11±4.08 *	7.7±6.2	10.8±5.5 *
Root hair number (between 5-10mm)	67.3±33.1	43.6±31.2	37.5±25.2 *	49.3±40.3
Root hair length (µm) (between 5-10mm)	243.24±47.79	209.13±47.7	226.27±38.22	228.46±33.14

Supplementary Table 1. Phenotypical parameters of transgenic *CcAOS* Arabidopsis plants.

Rosette area and dry weight were measured 1 month after germination. Time of flowering was measured 2 weeks after germination. The remaining root parameters were measured 13 days after germination. Measurements (means ± standard deviation) are from three independent assays, each using 5 plants per genotype. Asterisks refers to significant differences from the L*er*-0 wild type ($P<0.05$, *t*-test).

Gene name, locus and relation to pathway (JA – jasmonate; SA – salicylate)	Forward (5’-3’)	Reverse (5’-3’)	Melting temperature (ºC)
ALLENE OXIDE SYNTHASE AT5G42650.1 (JA)	CGACGGTGGGGAATAAACAA	TCGCCGGAAAATCTCAATCA	60
LIPOXYGENASE 1 AT1G55020.1 (JA)	GATGGGCTTGAGGTTTGGTA	TTCACGGGTTTGCATTTTAGG	62
JASMONATE-ZIM-DOMAIN PROTEIN 10 AT5G13220.1 (JA)	CGATTTCCTCGGACTTGAGA	GGAACCGAACGAGATTTAGC	60
PLANT DEFENSIN 1.2 AT5G44420.1 (JA) (Proietti et al., 2018)	CACCCTTATCTTCGCTGCTCTT	GCCGGTGCGTCGAAAG	58
PATHOGENESIS-RELATED GENE 5 AT1G75040.1 (SA)	ACTCCAGGTGCTTCCCGACA	ACTCCGCCGCCGTTACATCTT	60
NONEXPRESSER OF PR GENES 1 AT1G64280.1 (SA)	TTATCTGGCCGCCGAACAAG	AATCATCCGGCGAGTCAAAG	60

Supplementary Table 2. Primers used in RT-qPCR analysis of jasmonic acid and salycilic acid pathway genes.

```
              ....|....|....|....  |....|....|....|....  |....|....|....|....
                 5        15        25        35        45        55
Cc AOS        MASTSLAFP- SLQPKFQSSR KPSKPSTRRF IVRPITASVS EKPSVSVPPA TVVEQAEPTK
At AOS        MASISTPFPI SLHPKTVRS- ---KPLKFRV LTRPIKASGS E-----  TPDL TVATRTGSKD
Consensus     MAS S  FP  SL PK   S     KP   R    RPI AS S E       P   TV

              ....|....|....|....  |....|....|....|....  |....|....|....|....
                 65        75        85        95       105       115
Cc AOS        LPIKKIPGNY GLPFVGPIRD RFDFFYNQGR DEYFKSRAHK YQSTVFRANM PPGPLIASNP
At AOS        LPIRNIPGNY GLPIVGPIKD RWDYFYDQGA EEFFKSRIRK YNSTVYRVNM PPGAFIAENP
Consensus     LPI  IPGNY GLP VGPI D RDFYQG      E FKSR  K YSTVRNM    PPG  IA NP

              ....|....|....|....  |....|....|....|....  |....|....|....|....
                125       135       145       155       165       175
Cc AOS        NVVVLLDGKS FPVLFDVTKV EKKDLFTGTF MPSTELTGGY RVLSYLDPSE PNHGKLKRLL
At AOS        QVVALLDGKS FPVLFDVDKV EKKDLFTGTY MPSTELTGGY RILSYLDPSE PKHEKLKNLL
Consensus      VV LLDGKS FPVLFDV KV EKKDLFTGT  MPSTELTGGY R LSYLDPSE PHKLKLL

              ....|....|....|....  |....|....|....|....  |....|....|....|....
                185       195       205       215       225       235
Cc AOS        FFHLKARRDH VIPEFHSSYT ELFEGLENEL ATKGKAAFGE PSDLAAFNFL ARSLYGTNPV
At AOS        FFLLKSSRNR IFPEFQATYS ELFDSLEKEL SLKGKADFGG SSDGTAFNFL ARAFYGTNPA
Consensus     FF LK   R      PEF   Y ELF  LE EL    KGKA FG   SD   AFNFL AR  YGTNP

              ....|....|....|....  |....|....|....|....  |....|....|....|....
                245       255       265       275       285       295
Cc AOS        DTKLGLDAPK MIDKWLFVQI SPLFSLGLPK HLDDLLLRTV RLPPALVKAD YQKLYDFFYA
At AOS        DTKLKADAPG LITKWVLFNL HPLLSIGLPR VIEEPLIHTF SLPPALVKSD YQRLYEFFLE
Consensus     DTKL   DAP   I KW        PL S GLP        L   T   LPPALVK D YQ LY FF

              ....|....|....|....  |....|....|....|....  |....|....|....|....
                305       315       325       335       345       355
Cc AOS        SSGFVLDEAE RLGISREEAC HNLLFATCFN SFGGMKFLFP NMVKLIGRAG VKLHTQLAEE
At AOS        SAGEILVEAD KLGISREEAT HNLLFATCFN TWGGMKILFP NMVKRIGRAG HQVHNRLAEE
Consensus     S G   L EA   LGISREEA HNLLFATCFN   GGMK LFP NMVK IGRAG    H  LAEE

              ....|....|....|....  |....|....|....|....  |....|....|....|....
                365       375       385       395       405       415
Cc AOS        IRSVIRSNGG NITMAAMEQM PLMKSVVYES LRIEPPVALQ YGKAKKDLVI ESHDAAFRVK
At AOS        IRSVIKSNGG ELTMGAIEKM ELTKSVVYEC LRFEPPVTAQ YGRAKKDLVI ESHDAAFKVK
Consensus     IRSVI SNGG      TMAEM  L KSVVYE LR EPPV  Q YG AKKDLVI ESHDAAF VK

              ....|....|....|....  |....|....|....|....  |....|....|....|....
                425       435       445       455       465       475
Cc AOS        EGEMLFGYQP FATKDPKIFE RAEEFVADRF VGEEGEKLLK HVLWSNGPES ESPSVGNKQC
At AOS        AGEMLYGYQP LATRDPKIFD RADEFVPERF VGEEGEKLLR HVLWSNGPET ETPTVGNKQC
Consensus      GEML GYQP  AT DPKIF RA EFV  RF VGEEGEKLL  HVLWSNGPE  E P VGNKQC

              ....|....|....|....  |....|....|....|....  |....|..
                485       495       505       515       525
Cc AOS        AGKDFVVLVA RLLVVELFLR YDSIEIEVDS SPLGAALTVT SLKKASF
At AOS        AGKDFVVLVA RLFVIEIFRR YDSFDIEVGT SPLGSSVNFS SLRKASF
Consensus     AGKDFVVLVA RLVEFR     YDS  IEV  SPLG       SL KASF
```

Supplementary Figure 1. Alignment of amino acid sequences of *Castanea crenata* (CcAOS) and *Arabidopsis thaliana* (AtAOS) allene oxide synthase. Clustal W alignment was conducted using the BioEdit Sequence Alignment Editor. Numbers refer to the amino acid position.

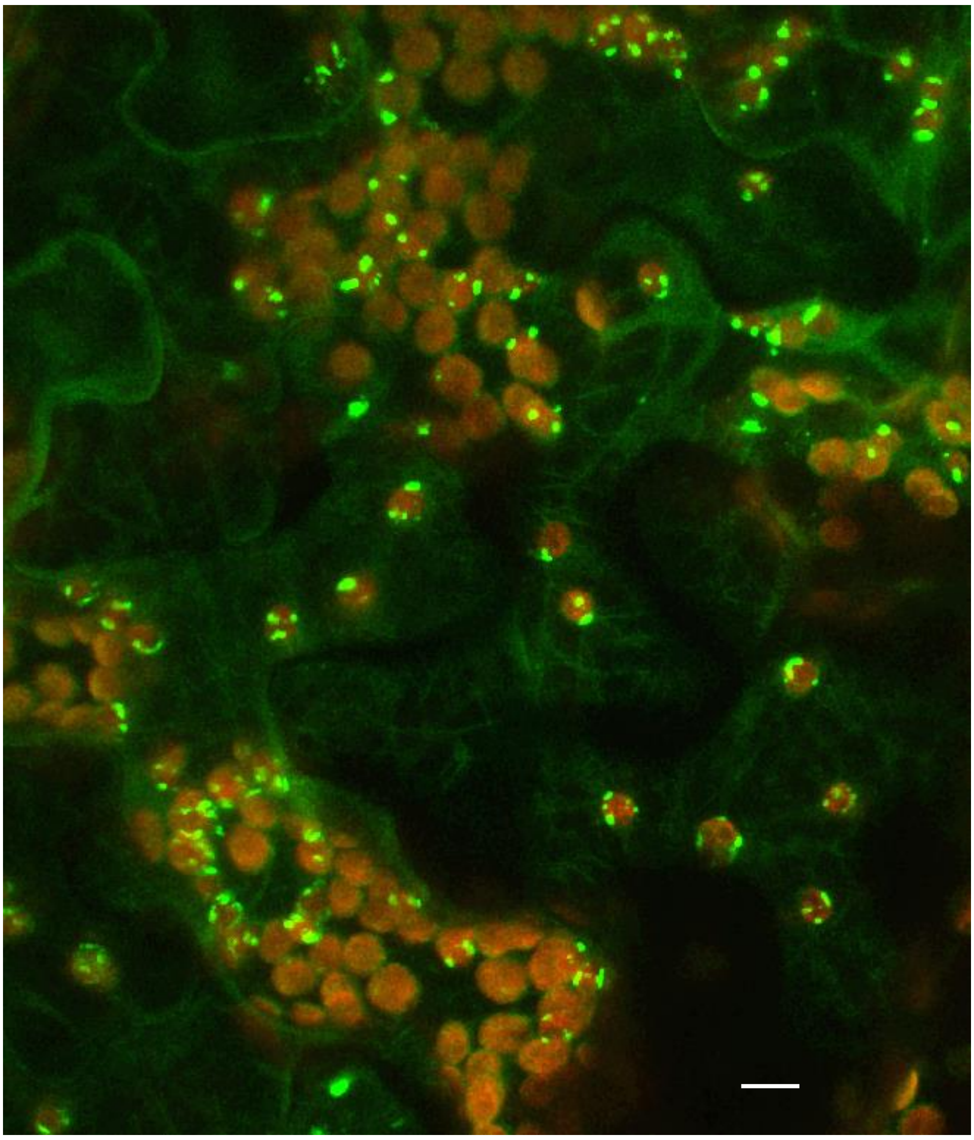

Supplementary Figure 2. 2D stack of subcellular imaging upon transient CcAOS-eGFP expression in leaves of *N. benthamiana*. Although CcAOS clearly accumulates at chloroplasts, a reticulate-like signal in the cytosol suggests that trafficking of the protein may involve the endomembrane compartment. Scale Bar = 10 µm.

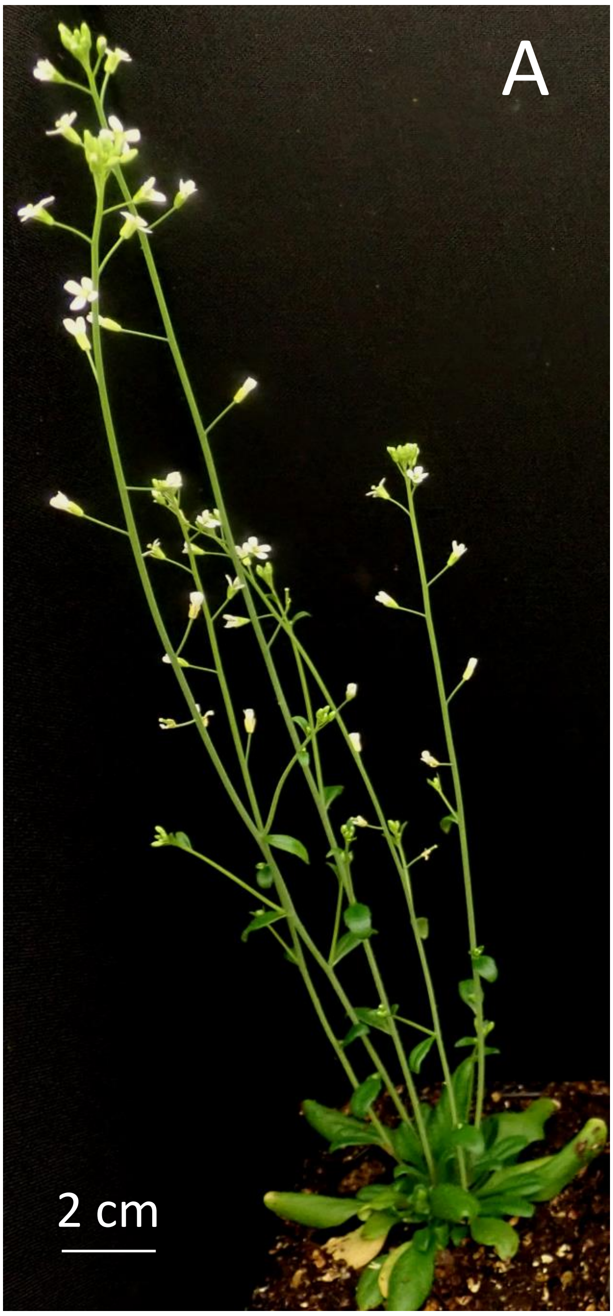

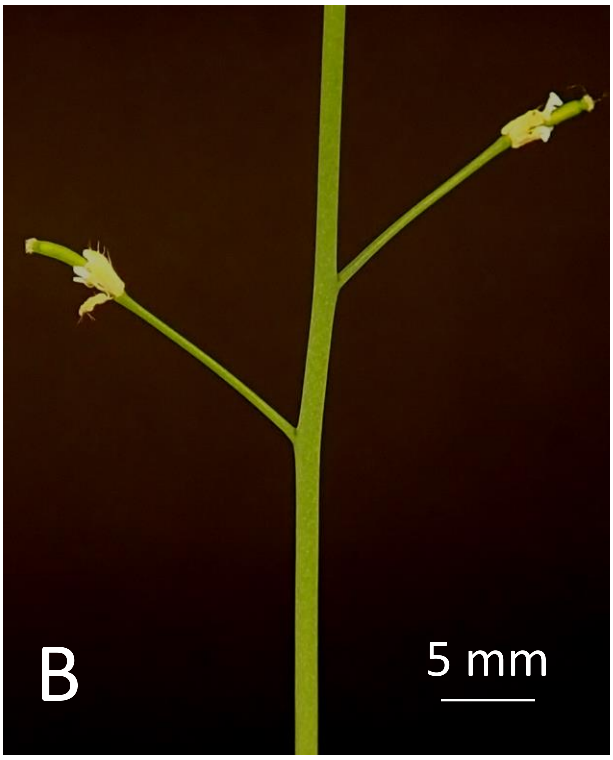

Supplementary Figure 3. Two-month old *Arabidopsis thaliana aos-GK624b02* mutant plant (A) and detail of siliques (B). The site of the T-DNA insertion in the mutant (5'UTR) is described in https://www.gabi-kat.de/db/visualization.php?lineid=624B02&genecode=At5g42650 and is also reported in https://www.arabidopsis.org/servlets/TairObject?id=503215774&type=polyallele

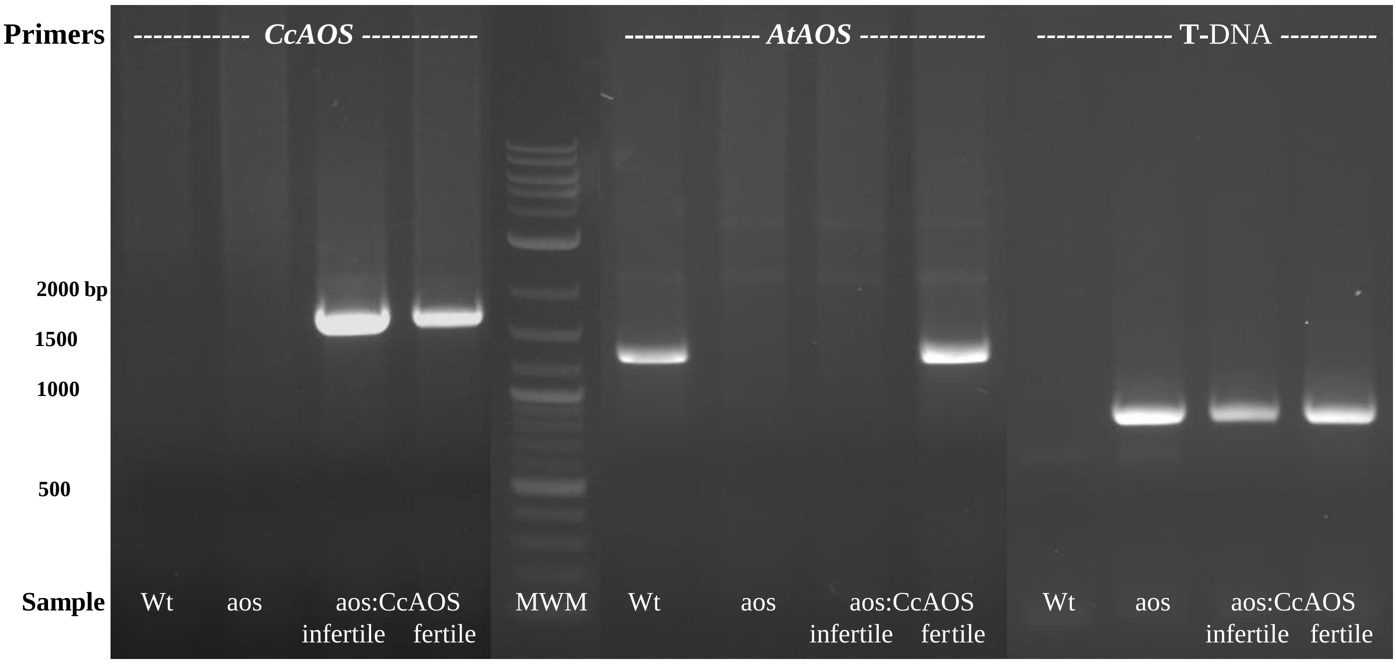

Supplementary Figure 4. Genotyping of plants after expression of *CcAOS* gene in *aos-GK624b02* mutants. Genomic DNA of F2 *aos:CcAOS*-infertile and *aos:CcAOS*-fertile plants was screened for *CcAOS*, *AtAOS* and T-DNA insertion and further compared with wild-type Col-0 (Wt) and *aos* plants (used as controls). The expected band sizes are 1581 bp for the amplification of *CcAOS* ORF, 1345 bp for the amplification of a fragment of non-disrupted *AtAOS* and 850 bp for the amplification of a fragment of the T-DNA left border fused to *AtAOS* (T-DNA). MWM: molecular weight marker N3200L (New England Biolabs, Ipswich, MA, USA).
